# Improving multi-trait genomic prediction using synthetic traits from hyperspectral data based on co-heritability

**DOI:** 10.1007/s00122-026-05334-2

**Published:** 2026-09-18

**Authors:** Ashmita Upadhyay, Ruhana Azam, Meilu Yuan, Stefan Ivanovic, John N. Ferguson, Rachel E. Paul, Sanmi Koyejo, Mohammed El-Kebir, Alexander E. Lipka, Andrew D. B. Leakey, Samuel B. Fernandes

**Affiliations:** 1 Department of Crop, Soil, and Environmental Sciences & Center for Agricultural Data Analytics, University of Arkansas, Fayetteville, AR 72701 USA; 2 Siebel School of Computing and Data Science, University of Illinois Urbana-Champaign, Urbana, IL 61801 USA; 3 Institute for Genomic Biology, University of Illinois Urbana-Champaign, Urbana, IL 61801 USA; 4 Department of Plant Biology, University of Illinois Urbana-Champaign, Urbana, IL 61801 USA; 5 Computer Science, Stanford University, Stanford, CA USA; 6 Department of Crop Sciences, University of Illinois Urbana Champaign, Urbana, IL 61801 USA

## Abstract

***Key message*:**

**Synthetic traits, wavelength ratios selected by co-heritability, raised multi-trait genomic predictive ability for leaf nitrogen and specific leaf area in sorghum by up to 17% over single-trait models.**

**Abstract:**

Genomic prediction (GP) is an essential tool in plant breeding as it can accelerate cultivar development by predicting the performance of unphenotyped lines. When using single-trait GP models, the precision of prediction is constrained by the heritability of the target trait. Multi-trait genomic prediction can be used to improve accuracy but requires identifying secondary traits with high heritability and genetic correlation with target traits. This study assessed the efficiency of multi-trait genomic prediction models powered by secondary traits derived from high-throughput phenotyping data when predicting leaf nitrogen content (N) and specific leaf area (SLA) in diverse sorghum accessions. We hypothesized that wavelength ratios from hyperspectral data could serve as synthetic secondary traits. Therefore, we developed models for direct measures of N and SLA, plus partial least squares regression (PLSR) predictions of them (Leaf N-PLSR, SLA-PLSR), totaling four target traits. Three synthetic traits (S1, S2, S3), each a ratio of two wavelengths within the hyperspectral data, were identified based on high co-heritability with target traits. Single-trait (genomic best linear unbiased prediction (GBLUP) served as the baseline model, followed by multi-trait GBLUP models combining synthetic and target traits. Model performance was assessed using fivefold cross-validation under single-trait, CV1, and CV2 schemes. Our approach improves multi-trait genomic prediction of target traits by using synthetic traits with no intrinsic biological meaning selected through co-heritability estimation. This demonstrates that there’s more useful information in the spectra than is typically utilized, and this information can be leveraged to improve multi-trait prediction of a target trait.

**Supplementary Information:**

The online version contains supplementary material available at https://doi.org/10.1007/s00122-026-05334-2.

## Introduction

Genomic prediction (GP) is an essential tool in plant breeding that reduces the time required to release a new cultivar. It thus contributes to improving resilient food, fuel, bioproducts, feed, and fiber production. The strength of GP stems from its ability to utilize the phenotypic and genotypic information from a training population to predict the performance of individuals that were not evaluated in field trials (Hickey et al. 2014). Thus, it can speed up the selection cycle, as well as allow a higher selection intensity (Xu et al. 2021). Examples of successful applications of GP include prediction of disease resistance in wheat (Azizinia et al. 2020), nitrogen concentration in maize (Barzin et al. 2021), yield prediction in wheat (Bian et al. 2022; Crain et al. 2018), soybean (Da Silva et al. 2020), maize (Fernandes et al. 2024), and rice (Grenier et al. 2015), and biomass in sorghum (Fernandes et al. 2018). However, the upper limit of the predictive ability is the square root of the heritability ($\sqrt{h^2}$) (Guo et al. 2014; Calus and Veerkamp 2011; Jia and Jannink 2012; Hayashi and Iwata 2013), which limits the usefulness of GP for traits of low $h^2$.

One approach that has been used to improve predictive ability in traits of low $h^2$ is multi-trait (MT) GP (Jia and Jannink 2012; Gaire et al. 2022; Gill et al. 2023; Fernandes et al. 2018; Kaler et al. 2022). Including a secondary trait in the model improves predictive ability when the secondary trait has a high $h^2$ and is highly genetically correlated with the target trait (Guo et al. 2014; Calus and Veerkamp 2011; Jia and Jannink 2012; Hayashi and Iwata 2013). However, MT requires prior identification of secondary traits with both high heritability and strong genetic correlation with the target trait. In the past, breeding programs were limited to phenotyping a few agronomic or morphological measures. However, with recent advances in remote sensing, robotics, and artificial intelligence-enabled analysis, it is now possible for a plethora of traits to be easily collected with high-throughput phenotyping (HTP) methods. Furthermore, hyperspectral data sets contain thousands of wavelengths, most of which remain underutilized in current applications. This study tests whether this untapped information in HTP data can be leveraged to ease the adoption of MT.

One commonly used modality of HTP data in plant science is hyperspectral reflectance spectra, which describe the intensity of reflected light at hundreds or thousands of wavelengths in the visible, near-infrared, and short-wave infrared portions of the electromagnetic spectrum (Krause et al. 2019). In many cases, a subset of wavebands are used to estimate some of the many vegetation indices that have been previously defined as useful proxies of vegetation form and function, e.g., normalized difference vegetation index (NDVI) (Da Silva et al. 2020; Aparicio et al. 2000), soil adjusted vegetation indices (SAVI) (Panda et al. 2010), or Normalized Difference Red Edge (NDRE). Alternatively, partial least squares regression models can be built to predict traits of interest from spectral data (Yendrek et al. 2017; Weber et al. 2012). Whichever approach is taken, there is commonly a significant amount of potentially useful information in the spectra that is collected but not utilized.

While GP and HTP data are independently powerful approaches, the optimum scenario would be an efficient integration of both tools. Attempts at this task have included obtaining a relationship matrix based on the HTP data (Krause et al. 2020) and utilizing previously validated indices as secondary traits in MT models (Sun et al. 2017). While improvements were observed in the former, in the latter, there is a bottleneck where it is necessary to validate that the index selected possesses the characteristics of a favorable secondary trait. As the requirement for secondary traits to be beneficial in MT revolves around correlation with the target trait as well as heritability, one potential option to summarise these measures is to calculate the co-heritability ($coh^2$) (Falconer 1996; Vasquez-Kool 2019). Therefore, we hypothesize that the $coh^2$ can be utilized to identify traits within HTP data that were not selected a priori or that do not have an intrinsic biological value but are powerful and flexible secondary traits for use in GP.

Hypothesis testing in this study uses leaf nitrogen content per unit area (Narea) and specific leaf area (SLA) of field-grown sorghum as a case study. Monitoring leaf nitrogen in the field is important for understanding many processes, including leaf metabolism, growth, ecosystem function, herbivory, litter decomposition, plant-microbe interactions, and fertilizer management (Melillo et al. 1982; Muchow and Sinclair 1994; Poorter and Evans 1998; Hamilton et al. 2001; Díaz et al. 2004; Uribelarrea et al. 2009; Sayer et al. 2017). Specific leaf area (SLA)-leaf lamina area per unit dry mass – is a key leaf trait tightly linked to variation in leaf composition, physiology, plant growth strategy, and resource investment (Shipley et al. 2006; Flores et al. 2014). Leaves with a high SLA typically have less nitrogen per unit leaf area, lower rates of photosynthesis, lower rates of respiration, are less well defended against herbivory, and decompose more rapidly than leaves with a low SLA (Wright et al. 2004).

## Materials and methods

In 2017, 869 photoperiod-sensitive sorghum lines were grown in two locations on the University of Illinois at Urbana-Champaign research farm, as described previously (Santos et al. 2020; Ferguson et al. 2021; Paul 2021). The experimental layout of each location was an augmented block design, with 16 incomplete blocks, and six check lines were included in each block. Each replicate field comprised 960 four-row plots of 3 m length, 1.5 m alley, and 0.76 m row spacing. Phenotyping was performed on the youngest fully expanded leaves of two randomly selected plants from the middle two rows of each plot as part of the sampling protocol described by Ferguson et al. (2021). The leaves were excised just above the ligule at dawn, and the cut end was immediately placed in water. In the laboratory, hyperspectral reflectance was measured at six locations on the adaxial surface in the central section of each leaf using a full-range spectroradiometer (ASD FieldSpec4™Standard-Res, Analytical Spectral Devices, Boulder, CO) equipped with an illuminated leaf clip. To conform with experiments on other species with smaller leaves, the leaf clip was fitted with a custom-made mask to limit illumination to an area of 150 $mm^2$. A reflective white reference disc (Spectralon, Labsphere Inc., North Dutton, NH) was used to standardize the relative reflectance of the samples. The instrument was turned on 30–60 min prior to use to allow the three arrays to warm up. Each spectrum collected was the internal average of 10 scans of the instrument, each taking  100 ms. Before averaging the measurements for a single leaf, a splice correction was applied to each spectrum to correct for the difference between the three sensors, and quality control was applied using the FieldSpectra package in R (Serbin et al. 2014). Subsequently, three 1.6 $cm^2$ leaf discs were collected from the same portion of the leaf where hyperspectral reflectance was assessed. Leaf discs of known area were immediately transferred to an oven set at 60 $^\circ$C for two weeks. SLA was calculated as the ratio of leaf area to dry mass. Leaf N content was determined using an elemental analyzer as described by Gray et al. (2012) after the leaf discs were pooled and powdered using a mechanical grinder (2000 Grinder, SPEX CertiPrep, Metuchen, NJ, USA). Partial least squares regression (PLSR) models were built to predict Narea and SLA (Paul 2021). These PLSR-based predictions of Narea (PLSR-Narea) and SLA (PLSR-SLA) were used alongside traditional measures of Narea and SLA as the four target traits in this study.

### Heritability estimation

The overarching hypothesis was that secondary traits derived from hyperspectral data could boost the predictive ability of target traits in multi-trait genomic prediction. Consequently, we sought to identify ratios of wavelengths within spectra with (i) the highest heritability and (ii) a high genetic correlation with the target trait of interest, i.e., Narea, SLA, PLSR-Narea, and PLSR-SLA. We focused on these two conditions because the resulting wavelength ratios exhibiting these properties were theoretically best suited for multi-trait models (Mrode 2014). Accordingly, the first step towards identifying these best-suited ratios was to estimate the heritability of the traits collected from these two locations. For that, we used the following linear mixed model:

1 $$\begin{aligned} y = X\beta + Z_{1}b + Z_{2}g + Z_{3}{gl} + e, \end{aligned}$$

where y is the vector of phenotypes; *X* is the incidence matrix associated with the vector of fixed effects β, which includes set and location; $Z_{1}$ is the incidence matrix associated with the vector of random effect of block *b* within set *s* and location *l*, with *b* ∼N(0, $\sigma ^2_{b}$); $Z_{2}$ is the incidence matrix associated with the vector of random effect of genotypes *g*, with *g* ∼ N(0, $\sigma ^2_g$); $Z_{3}$ is the incidence matrix associated with the vector of random effect of genotypes by locations interaction, with *gl* ∼ N(0, $\sigma ^2_{gl}$); and *e* is the vector of residuals, with *e* ∼N(0, $\sigma ^2_{e}I$). The terms $\sigma ^2_{b}$, $\sigma ^2_{g}$, $\sigma ^2_{gl}$, and $\sigma ^2_{e}$ are the variances of block within the set and location, genotypes, genotypes by locations, and residuals. Finally, *I* denotes the identity matrix. Model 1 was fitted in R (R Core Team) using the lme4 package (Bates 2014). Model 1 was used to estimate variance components for the four target traits evaluated (Narea, SLA, PLSR-Narea, PLSR-SLA). Because most indices utilized are linear combinations such as ratios of wavelengths, we investigated ratios of pairs of wavelengths (e.g., 400 nm / 2100 nm) (Lamb 1999; Wiegand et al. 1991; Thenkabail et al. 2000a; Khan et al. 2018). The computational workflow for this stage was implemented in the R package SyntheticTraits version 1.0.0 (https://github.com/fernandes-lab/SyntheticTraits), and it was executed trait-wise across the 350–2500 nm spectral window. This produced 4,626,801 candidate wavelength ratios per target trait, which were written to trait-specific output tables for downstream filtering. Once the variance components were obtained for the target traits and the wavelength ratios, the sample heritability ($\hat{H}^2$) was estimated using the equation below:

2 $$\begin{aligned} \hat{H}^2 = \frac{\hat{\sigma }^2_{g}}{(\hat{\sigma }^2_{g} + \frac{ \hat{\sigma }^2_{gl}}{l} + \frac{\hat{\sigma }^2_{e}}{lr})}, \end{aligned}$$

in which *l* is the number of locations, *r* is the harmonic mean of the number of replications, and all other terms are estimates of the parameters defined in model 1. Because this stage involved fitting millions of models, we focused on computational efficiency and used a simple heritability estimator, but more robust ones are available (Piepho and Mohring 2007).

### Co-heritability estimation and identification of synthetic traits

We next sought to determine which particular wavelength ratios were best suited to include as additional response variables in multi-trait genomic prediction. Such ratios were called synthetic traits, and these exhibited the strongest co-heritabilities with the target trait. The co-heritability between two traits is defined as their heritable genetic correlation and was calculated as follows (Falconer 1996):

3 $$\begin{aligned} \hat{coh^2_{TS}}= \hat{r}_{g_{(T,S)}} \times \sqrt{\hat{H}^2_{T}} \times \sqrt{\hat{H}^2_{S}}, \end{aligned}$$

where $\hat{coh^2_{TS}}$ is the co-heritability between the target and the synthetic (i.e., secondary) trait, $\hat{r}_{g_{(T,S)}}$ is the genetic correlation between the target and the synthetic trait, and $\sqrt{\hat{H}^2_{T}}$ and $\sqrt{\hat{H}^2_{S}}$ are the square root of sample heritability for the target trait and synthetic trait, respectively. To estimate the genetic correlation, we utilized the property of the variance of the sum of two random variables. First, we fitted a mixed linear model (1) to estimate the genetic variance for the target trait (i.e., Narea, SLA, PLSR-Narea, or PLSR-SLA), which was referred to as $\sigma _g$ in model 1, but to be more clearly associated with the target we will call it $\sigma _T$ and the genetic variance for the wavelength ratio will be called $\sigma _S$ to be associated with the synthetic trait. Second, we estimated the variance component for the target trait plus the wavelength ratio (e.g., $N + (Wave_{1000}/Wave_{200})$). In this case, we estimated the genetic covariance of the two variables:

4 $$\begin{aligned} {\hat{\sigma _T\sigma _S}} = \frac{({\hat{\sigma }^2_{(T+S)}} - {\hat{\sigma }^2_{T}}- {\hat{\sigma }^2_{S}})}{2}. \end{aligned}$$

Using these estimates of genetic variances and covariance, we next estimated the genetic correlation between *T* and *S* as follows:

5 $$\begin{aligned} \hat{r}_{g_{(T,S)}}= \frac{\hat{\sigma _T\sigma _S}}{\sqrt{\hat{\sigma }^2_T} \sqrt{\hat{\sigma }^2_S}}. \end{aligned}$$

To minimize the effect of possibly instable estimates of genetic correlation, the Pearson’s correlation of BLUPs (Best Linear Unbiesed Predictor) of the target and synthetic trait was used as a second estimator of genetic correlation, both estimates were saved for the downstream selection of the synthetic traits. The $\hat{coh^2_{TS}}$ calculation was implemented in the *getCoh*2() function of the SyntheticTraits package.

For each of the four target traits, 4,624,650 co-heritability values were estimated, one for each wavelength ratio, i.e., pairwise ratios of all 2151 bands excluding the ratios of a wavelength with itself. Heatmaps for each trait were generated to visualize the co-heritability across all traits, which can be found in Supplementary file 1 (see Appendix). Since this pipeline relies on linear mixed models, several ratios did not reach convergence and therefore a co-heritability estimate was not available for them.

For downstream analysis, we filtered out (i) ratios that did not converge; (ii) ratios for which the magnitude of the two estimates of genetic correlation (i.e., the estimate based on covariance and the Pearson’s correlation of BLUPs described above differed in more than 0.2; (iii) ratios with estimates outside of the parametric space (e.g., $\hat{r}_{g_{(T,S)}} > 1$ or $\hat{r}_{g_{(T,S)}} < -1$). To select a manageable number of synthetic traits for multi-trait genomic prediction, we selected the 1% wavelength ratios with the greatest co-heritability (top_pct = 0.01). Next, we calculated the Pearson correlation and Euclidean distance between these selected wavelength ratios. These Euclidean distances were subsequently used in a hierarchical cluster analysis to determine the extent to which these selected wavelength ratios clustered together. Because the multi-trait genomic prediction pipeline is time consuming, we decided to evaluate only three synthetic traits per target trait. As such, we identified three groups in the hierarchical cluster analysis. From each cluster we selected the traits with highest absolute value of $\hat{coh^2_{TS}}$ (i.e., $|\hat{coh^2_{TS}}|$). The selection of synthetic traits was implemented in the getTraits() function in the SyntheticTraits package. This procedure was repeated once for each target trait (i.e., Narea, SLA, PLSR-Narea, and PLSR-SLA).

For each target trait, a synthetic trait with co-heritability of zero was also selected as a control against which to assess any possible improvement in performance when utilizing highly co-heritable traits in the multi-trait framework. It is important to mention that the threshold of 1% was selected because we needed to drastically reduce the number of synthetic traits, but any other threshold could have been used.

The functions getCoh2() offers support for parallelization. Thus, to obtain the co-heritability estimates, we utilized 22 nodes with 96 cores (2112 total CPUs) with peak of memory of only 0.70 GB per core. The runtime to calculate the 4,624,650 co-heritabilities was around 4 h per trait (e.g., 3.97 h for Narea). All the results were plotted using the package ggplot2 (Wickham 2011).

### Genotypic data

The genotypic data set used in this study was previously published in Ferguson et al. (2021). Briefly, a set of 100,435 SNPs from Santos et al. (2020) was imputed (Beagle 4.1) using the whole-genome resequencing data set of 5,512,653 SNPs published in Valluru et al. (2019) as a reference. The resulting data set consisted of 5.5 million SNPs. This data set was filtered to remove SNPs with low imputation quality ($R^2 < 0.5$), markers with minor allele count less than 20 (vcftools), and SNPs with linkage disequilibrium higher than 0.9 in a window of 50 SNPs (Plink). This resulted in a total of 454,393 markers scored in 836 sorghum lines, which were considered in the ensuing analyses.

#### Genomic prediction

Genomic prediction was implemented in two stages. First, for each location (EF and MW), we obtained the best linear unbiased estimators (BLUEs) for target and synthetic traits using linear mixed models implemented in ASReml-R (Butler et al. 2017). In this first stage, BLUEs were estimated separately for each location and then combined across locations into a single analysis file per target trait, retaining the focal trait together with selected synthetic trait ratio(s). The statistical model for BLUEs estimation was as follows:

6 $$\begin{aligned} y = X \beta + Z_{1}b + e \end{aligned}$$

*y* is the phenotypic observation of the selected synthetic and target trait; *X* is the incidence matrix associated with the vector of fixed effect β, which includes genotype *g* and set *s*; $Z_{1}$ is the incidence matrix associated with the vector of random effect of block *b* with b ∼ N(0,$\sigma ^2_b$); and $e_{ijk}$ is the random effect of residuals, with *e* ∼ N(0, $\sigma ^2_{AR_{1}\times AR_{1}}$ ), where $AR_{1} \times AR_{1}$ is a first-order auto-regressive structure applied to rows and columns for spatial correction.

Next, we fitted the following genomic prediction model as a second-stage model using BLUEs from the first stage for a single trait:

7 $$\begin{aligned} Y = 1\mu + g + \varepsilon \end{aligned}$$

where Y is an n×t matrix of BLUEs of genotype effects from Model 6 estimated for each of t traits across n individuals; μ is a t-dimensional vector of grand mean parameter values for each of t traits, *g* is an n×t matrix of genetic effects for each of t traits following a matrix-variate normal distribution denoted g ∼ $N_{(n\times t)}$(0, $C_{g}$ ⊗ G$\sigma ^2_{g}$) with $C_{g}$ denoting the genetic variance-covariance between traits, *G* denoting the additive genetic relatedness matrix (VanRaden 2008), and $\sigma ^2_{g}$ denoting the genetic variance parameter; and *e* is an n×t matrix of error terms *e* ∼ $N_{(n\times t)}$(0, $C_{e}$ ⊗ I$\sigma ^2_{e}$) with $C_{e}$ denoting the non-genetic variance-covariance between traits, *I* denoting the identity matrix, and $\sigma ^2_{e}$ denoting the error variance parameter. In this study, n = 822 sorghum lines with genotypic information, t = 1 for the single-trait model that included only one of the four target traits, and t = 2 for the models that included one of the four target traits and one corresponding synthetic trait. Additional multi-trait variants were also evaluated with t=3 (target trait + two synthetic traits) and t=4 (target trait + three synthetic traits).

### Cross-validation scheme

The usefulness of synthetic traits to boost predictive ability in genomic prediction was assessed through *k*-fold cross-validation (CV), with *k* = 5 (Kohavi and John 1995). We aimed to utilize 25 replications of the 5-fold procedure, but because some replications did not converge, we run 25 replications and reported results on the first 20 reps that converged. Following the procedure described in Fernandes et al. (2018), three distinct scenarios were considered:

#### Single-trait (ST)

**Fig. 1:**
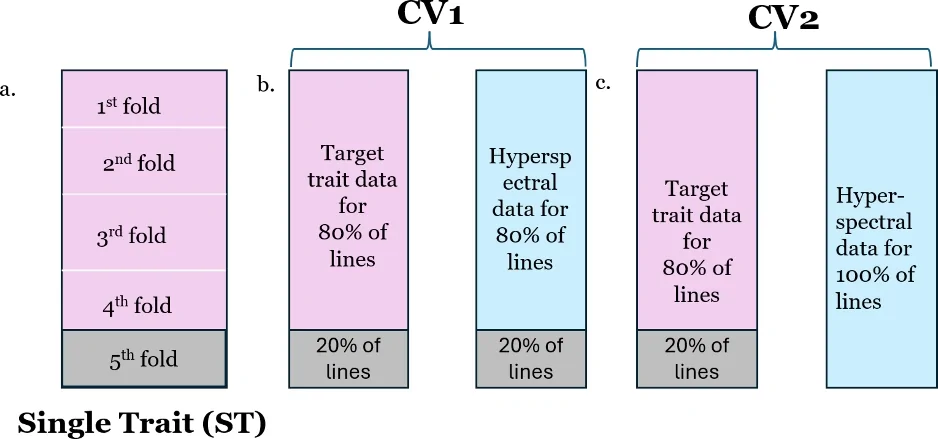
Diagrammatic representation of cross-validation scheme performed in single-trait and multi-trait genomic prediction model where only the target trait’s phenotypic data were used in ST scheme; however, both phenotypic data, i.e., target trait and hyperspectral data, are used for CV1 and CV2. In all cases, the training set utilized data from one location whereas the validation set utilized data from other location

Phenotypic values of a single target trait on 80% of the genotypes were used for training a single-trait GBLUP model to predict genomic estimated breeding values (GEBVs) of the target trait for the remaining 20% of individuals for validation (Fig. 1). Here, a single-trait GBLUP model, described in (8), was fitted in the training set with the target trait as the response variable. Predictive ability was evaluated using the Pearson correlation coefficient between the observed target trait value and the corresponding GEBV from the single-trait GBLUP model fitted in the training set.

#### CV1

Phenotypic data on the target trait and one of the corresponding synthetic traits from 80% of the genotypes were used to predict the GEBVs of the target trait for the remaining 20% of the genotypes. In this scenario, the multi-trait GBLUP model, described in (8), was fitted in the training set with the target trait and one of the synthetic traits as the two response variables. For test-fold genotypes, both the target trait and synthetic trait observations were masked when fitting CV1 models. Predictive ability was evaluated using the same metric described in the ST scenario.

#### CV2

Also known as trait-assisted (Fernandes et al. 2018), the CV2 scheme utilized phenotypic data on the target trait from 80% of the individuals and phenotypic data on one of the corresponding synthetic traits from 100% of genotypes to predict the GEBVs of the target trait for the remaining 20% of genotypes. Here, the multi-trait GBLUP model, described in model 7, was fitted to all genotypes for the target trait and the synthetic trait in the training set, as well as to all genotypes for the synthetic trait in the validation set. For test-fold genotypes, only the target trait was masked, while synthetic trait observations were retained. The same metric described in the ST scenario was used to evaluate predictive ability.

The same folds were used to assess performance across the three scenarios to enable a thorough comparison of prediction accuracies. To minimize the possibility of overfitting and obtain an over-optimistic assessment of model performance, as described in Runcie and Cheng (2019), we used one location for training and another location for validation in directional transfer scenarios (EF→MW and MW→EF). For each scenario and repetition, accuracies and predictions were written to per-repetition files and subsequently aggregated for summary analyses.

### Control measure

A control measure was taken to validate the idea of selecting synthetic traits that were highly heritable and genetically correlated with the target trait. The opposite was also done, where one synthetic trait with a co-heritability value of zero was selected for each target trait and used to fit the multi-trait genomic prediction model; model performance was assessed using both CV1 and CV2 schemes under the same cross-validation partitions.

### 20:80 approach

**Fig. 2:**
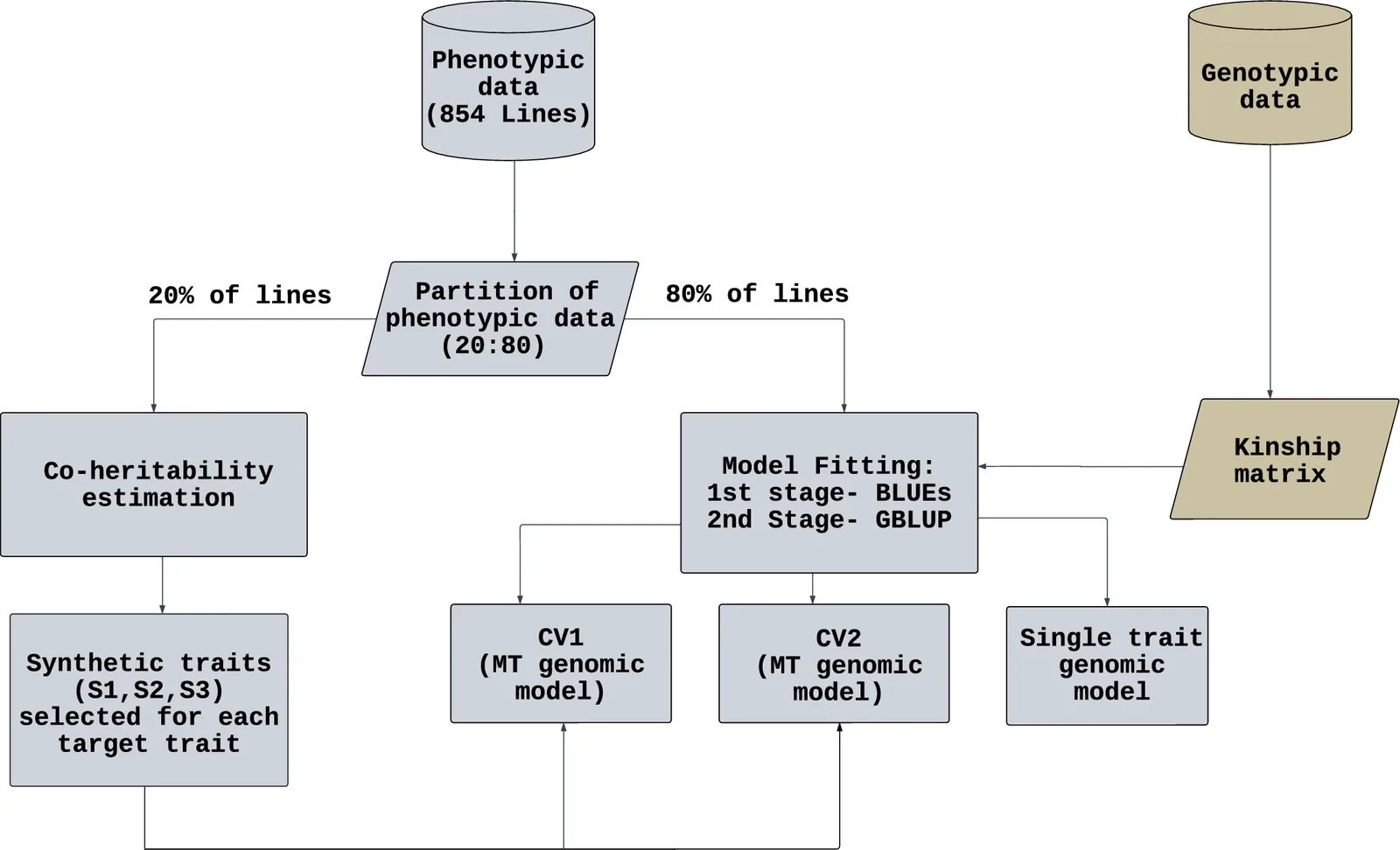
Flow chart with the methods used to separate 20% of the individuals from the whole data, estimate the co-heritability, select three synthetic traits, and use the selected synthetic trait further in a multi-trait (MT) genomic prediction model fitted for 80% of the data for four target traits

We conducted an additional analysis to explore the extent to which using the same individuals to identify synthetic traits increased the predictive ability of our multi-trait GBLUP models. Specifically, a testing framework was developed to identify synthetic traits on a subset of 20% and run genomic prediction on the remaining 80% as described in Fig. 2. Thus, the individuals were randomly split in to five folds of 20% and each fold was used at a time to identify synthetic traits using model 3 similar to a typical 5-fold cross validation scheme. Thus, running GP on subset 1 meant using fold 1 for co-heritability estimation and folds 2-5 (remaining 80% individuals) for genomic prediction. Similarly, running GP on subset 5 meant identifying synthetic traits on subset 5 and using the remaining folds 1-4 for genomic prediction. Model performance was assessed using the cross-validation schemes CV1, CV2, and the one described in ST.

## Results

**Fig. 3:**
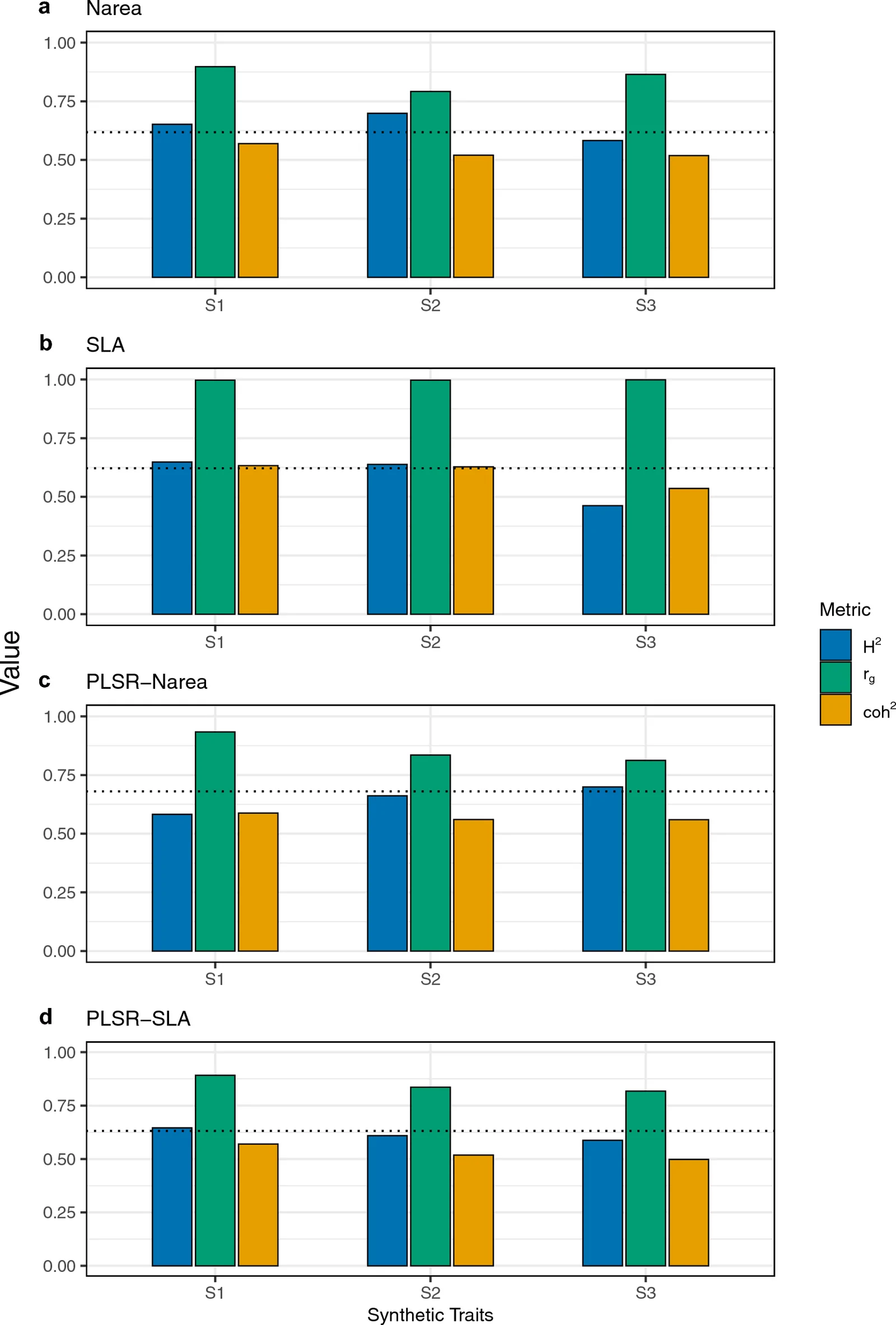
Observed heritability ($H^2$), absolute value of genetic-correlation ($r_g$), and co-heritability ($coh^2$) of synthetic trait. The X-axis (Synthetic Traits) consists of S1, S2, and S3 across four target traits and twelve ratios, where three were selected for each trait, and the Y-axis consists of the value of the three different metrics observed. The target trait’s heritability is the black dotted line in each sub-figure a, b, c, and d

Four target traits were used as case studies, and for each one, we identified three synthetic traits (S1, S2, S3) involving different wavebands (Table S1) to be included in the multi-trait genomic prediction models. Thus, we identified 12 different synthetic traits. Each trait was used in bivariate models with the respective target trait, as well as in a combined model with the target trait and all three synthetic traits simultaneously (S123). The target traits utilized here had heritabilities of 0.61, 0.62, 0.68, and 0.63 for Narea, SLA, PLSR-Narea, and PLSR-SLA, respectively (Fig. 3). The synthetic traits had heritability ranging from 0.46 to 0.70. The absolute value of $\hat{r}_{g_{(T,S)}}$ ranged from 0.71 to 0.99. Finally, the $\hat{coh^2_{TS}}$ for the 12 synthetic traits ranged from 0.50 to 0.63.

### Utilizing synthetic traits in MT-GP can boost predictive ability compared to ST models

**Fig. 4:**
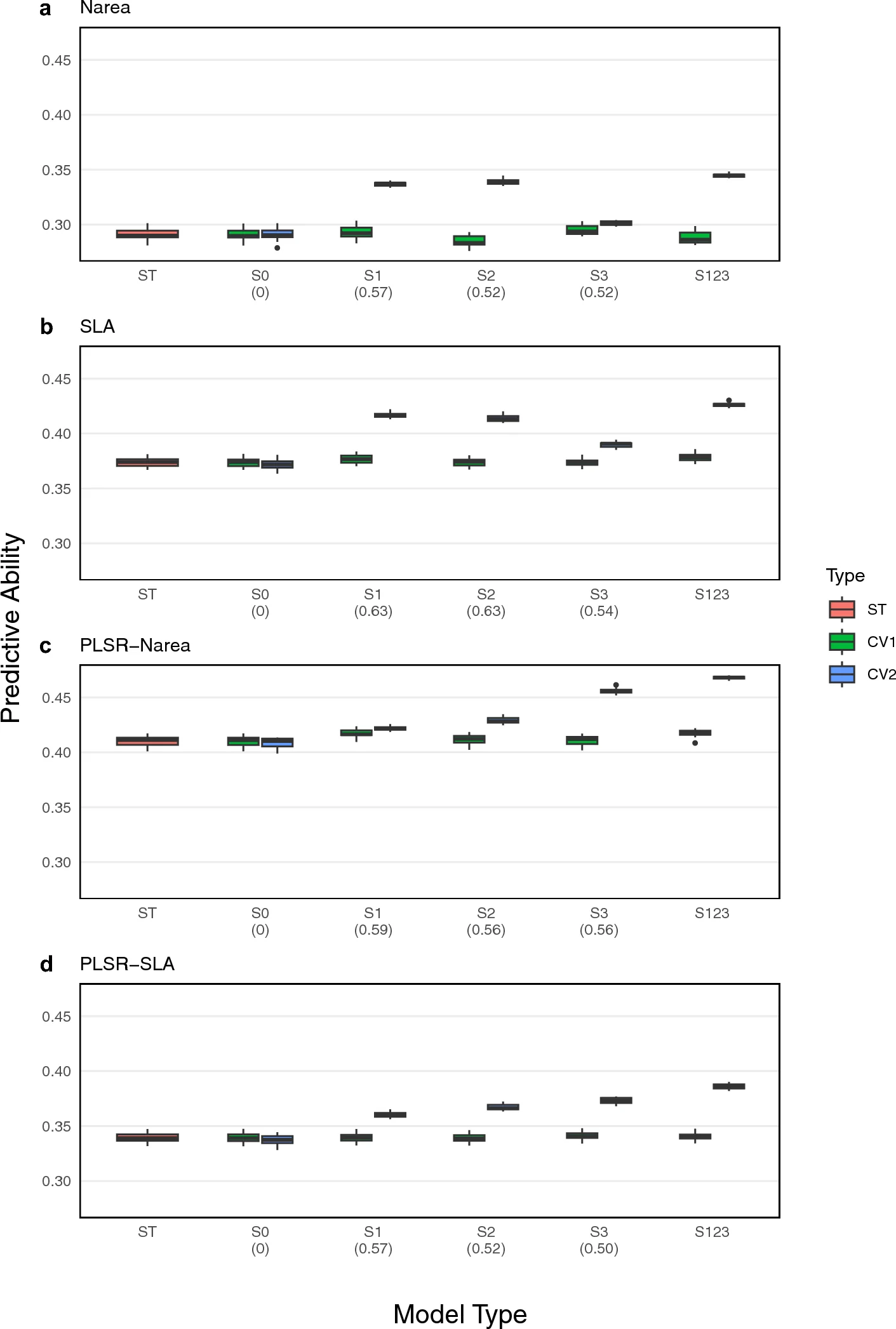
Single-trait and multi-trait genomic prediction models for four target traits: Narea, SLA, PLSR-Narea, PLSR-SLA. The Y-axis represents the predictive ability for single trait (ST), multi-trait prediction utilizing a synthetic trait selected with zero co-heritability (S0), Synthetic Trait 1 (S1), Synthetic Trait 2 (S2), Synthetic Trait 3 (S3), and a multi-trait model with the target trait and the three synthetic traits simultaneously. The annotations on the x-axis below the synthetic trait are the co$h^2$ value for each synthetic trait. The model was trained using the data set from Energy Farm (EF) and validated on the data set from Maxwell Farm (MW). The blue boxplot is the distribution of accuracies for 20 replications for CV2, the green box plot is for CV1, and the red color is for the single-trait model

We selected three synthetic traits of high $coh^2_{TS}$ and used them to fit multi-trait genomic prediction models. The predictive ability of the multi-trait models, the CV2 in particular, was consistently higher than that of the single-trait models resulted in the highest predictive ability in all cases (Fig. 4). The CV1 performed similarly to the single-trait model. Overall, the predictive ability increased from 0.29 to 0.34 for Narea, 0.37 to 0.42 for SLA, 0.41 to 0.45 for PLSR-Narea, and from 0.34 to 0.37 for PLSR-SLA when comparing single-trait to multi-trait models. That represents an increase of approximately 17.24% for Narea (Fig. 4a), 13.51% for SLA (Fig. 4b), 9.75% for PLSR-Narea (Figure 4c), and 8.82% for PLSR-SLA (Fig. 4d), compared to ST. The multi-trait model including the target and all the respective synthetic trait resulted in a predictive ability at least as high as the best bivariate model, except when predicting PLSR-SLA using MW as the training set (S6).

### Synthetic traits with lowest co-heritability did not improve prediction compared to single trait model

The idea of selecting synthetic traits based on their $coh^2_{TS}$ value was further investigated, so we fitted the model using a synthetic trait with the lowest $coh^2_{TS}$ (S0) value to check its usefulness. Overall, the synthetic trait S0 when utilized alongside the target trait did not result in any improvement in prediction (Fig. 4). In general, the performance was similar to the single-trait model for both CV1 and CV2 schemes. In Fig. 4 for Narea, the average predictive ability of a single trait and MT was equal, i.e., 0.29, for SLA the predictive ability of both ST and MT models was also equally ranged with an average predictive ability of 0.37 for both models, for PLSR-Narea the predictive ability of ST and MT was 0.41, and for PLSR-SLA, the predictive ability of both models was 0.34. The synthetic trait with $coh^2_{TS}$ value zero selected for Narea had $H^2$ of 0.40, for SLA, the $H^2$ was 0.37, for PLSR-Narea it was 0.43, and for PLSR-SLA the $H^2$ was 0.51, with $\hat{r}_{g_{(T,S)}} = 0$ for all four target traits. In summary, the factor common in all synthetic traits with $coh^2_{TS}$ zero was the lowest genetic correlation with the target trait. Thus, the synthetic trait with co-heritability zero did not improve the predictive ability of the multi-trait model compared to the single-trait model.

### Using only 20% of hyperspectral data is enough to improve predictive ability

**Fig. 5:**
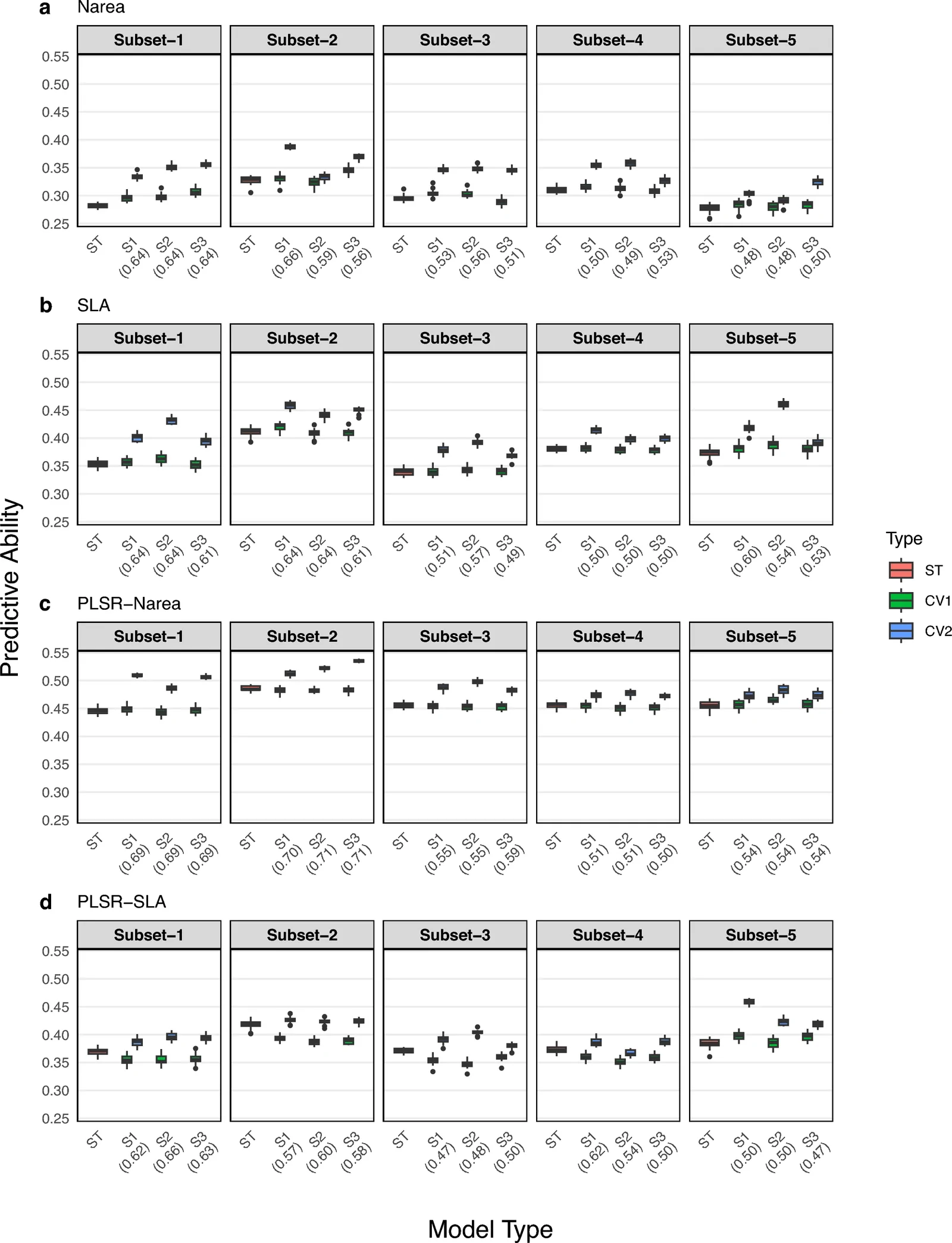
Comparison of multi-trait (MT) and single-trait (ST) models using synthetic traits identified from subsets of 20% of the individuals. The Y-axis is the predictive ability of models, and the X-axis is the type of model used, i.e., MT and ST. The blue color boxplot is the distribution of predictive ability of 20 replications for CV2, the green color boxplot is for CV1, and the red color is for a single trait. The numbers in parenthesis are the co-heritability values for each synthetic trait. The model was trained using the data set from Energy Farm and validated on the data set from Maxwell Farm

To investigate whether or not the use of the same individuals to identify synthetic traits and to train the genomic prediction models creates a problem, we compared the predictive ability trend of the complete model with five mutually exclusive subsets. The trend of predictive ability when utilizing only 20% of the individuals to identify the synthetic trait was very similar to the observed when utilizing the full data set (Fig. 5).

In this case, the predictive ability of the best subset MT model was boosted from 0.30 to 0.34 for Narea, 0.37 to 0.42 for SLA, 0.46 to 0.49 for PLSR-Narea, and 0.38 to 0.41 for PLSR-SLA across the five subsets within CV1 and CV2. In summary, the subset’s MT model predictive ability was increased by 15.61% for Narea, 14.24% for SLA, 7.44% for PLSR-Narea, and 6.98% for PLSR-SLA in Fig. 5a, b, c and d compared to ST model. As expected, the numeric values of predictive ability were greater when utilizing the complete set of individuals, but the comparisons across models were similar.

## Discussion

In this study, we generated a set of four million wavelength ratios that, as a trait set, describe a substantial fraction of the potential information that is present in a hyperspectral HTP data set. The set of wavelength ratios was filtered using the $coh^2$ measure to identify secondary traits for MT-GP models. Thousands of ratios were selected within the top 1%, but since MT-GP models are computationally demanding, we selected an arbitrarily manageable number (i.e., three) of what we called synthetic traits for each target trait considered. We hypothesized that the $coh^2$ would allow us to select synthetic traits to increase multi-trait predictive ability. The results supported this hypothesis, with MT-GP models consistently outperforming single-trait GP models for all four target traits. Furthermore, we demonstrated that the improvement in predictive ability was only present when the synthetic trait was highly co-heritable. Overall, our analysis highlights an effective approach to leverage high-throughput phenotyping data to improve the performance of genomic prediction models.

### High co-heritability was an indication of high predictive ability

Multi-trait models can leverage the correlation between traits to improve model fitting and predictive ability Mrode (2014). Furthermore, heritability has long been discussed as one of the main factors in delimiting predictive ability in genomic prediction Combs and Bernardo (2013). In this study, we demonstrated that co-heritability can be efficiently used to select secondary traits for multi-trait prediction. Similar to what have been observed in studies utilizing traits with an intrinsic biological value as secondary traits (Gill et al. 2023; Lado et al. 2018; Fernandes et al. 2018; Jannink et al. 2010), synthetic traits with no intrinsic biological value but with high co-heritability with the target trait, were able to improve the target trait’s predictive ability. This illustrates the potential information hidden in HTP data. While we only picked the top three synthetic traits, this number was arbitrary, and because all of them performed similarly, we can anticipate that many other synthetic traits could be identified from the millions of wavelength ratios obtained.

Additionally, in this study, the synthetic trait with lower co-heritability, i.e., zero, which in this case meant low genetic correlation between two traits, did not show any evidence of boosting target trait predictive ability when used in the MT-GP model, which aligns with the result in the study done by Krause et al. (2020). In that case, the use of a biologically meaningful secondary trait with low heritability and genetic correlation resulted in poor performance of MT-GP model.

### Important information can be mined from hyperspectral data

Hyperspectral data have been successfully utilized in GP before to calculate vegetation indices (Crain et al. 2018; Rutkoski et al. 2016; Moreira et al. 2020). However, we demonstrate here that only using predefined vegetation indices calculated from hyperspectral data likely fails to deliver the full potential value of hyperspectral data. Our results illustrate that wavelength ratios of high co-heritability can efficiently improve predictive ability regardless of being associated with a biological trait. As many indices are linear combinations of wavelength bands (Baret and Guyot 1991; Qi et al. 1993), for simplicity, we opted to investigate ratios of individual wavelengths. Nonetheless, more complex spectral combinations should also be explored in the future as this method is refined.

PLSR models have proven to be useful in handling high-dimensional hyperspectral data, taking control of multi-collinearity, and providing robust, reliable measures for the prediction of traits (Metz et al. 2021; Xu et al. 2018). In this study, we utilized PLSR predictions from a model previously validated by Paul (2021) to predict Narea and SLA based on hyperspectral information, i.e., PLSR-Narea and PLSR-SLA. The heritability of the PLSR traits was very similar to that of the biological traits, and when combined with the synthetic trait in the multi-trait framework, the predictive ability for the two traits was even higher than the respective biological traits, Narea and SLA. It is important to note that PLSR predictions were obtained from the same biological traits and hyperspectral data utilized for co-heritability estimation and genomic prediction, which might contribute to overly optimistic results for PLSR models. Nevertheless, this does not invalidate the trend of boost in predictive ability when comparing single trait models especially with the CV2 scenario. While it is evident that any prediction model relies on ground truth data for validation, this indicates a potential path to accuratly predict traits such as Narea and SLA with limited requirements for slow traditional measurement methods. In addition, the use of the hyperspectral data is very scalable because it allows analysis to be performed on multiple target traits (e.g., leaf N, SLA, photosynthetic capacity, sucrose content, disease status, etc) without any additional measurement effort. For plant breeding, these results reinforce the importance of HTP platforms, especially for physiologically important leaf traits that can be predicted based on spectral information.

### Training set composition affects the MT-GP performance

The composition of the training set plays a critical role when determining the end result of many GP models (Clark et al. 2019). The GP model is trained using the training population marker and phenotype data, which later predict the breeding values of the test set (Meuwissen et al. 2001). In Fig. 5a–d, the model fitted on the second subset consistently performed better than when other subsets were used, regardless of the target trait we considered. The sorghum diversity panel consists of different sorghum populations, which suggests the potential reason behind the subset of genotypes performing differently when used for model fitting compared to other subsets. Unlike when using the whole data to identify the synthetic trait and fit the GP model, with subset 2, the synthetic traits were identified from random 20% of lines, and the GP model was fitted with the remaining 80%, where the performance of the MT-GP model using the subset 2 was better in all four traits. This suggests that training set composition has an impact on the predictive ability of the GP model as previously observed (Olatoye et al. 2020).

Furthermore, the CV2 scheme, also known as trait-assisted in the multi-trait context, consistently produced higher prediction accuracies than the CV1 and ST models. The CV2 model is trained by 80% of the target trait data, and 100% of hyperspectral data, where adding hyperspectral information significantly boosted the predictive ability. This result is in accordance with several other instances that show that the CV2 is able to borrow information from the correlation between the secondary trait for which the additional 20% information is available (Fernandes et al. 2018; Burgueño et al. 2012; Guo et al. 2014).

### Synthetic traits included meaningful spectral regions

As emphasized, the synthetic traits identified do not necessarily have an intrinsic biological value. However, a subset of the wavelengths involved in those traits align with wavelengths that feature in commonly used vegetation indices (Xue 2017; Sims and Gamon 2003; Thenkabail et al. 2000b). For example, the near infrared waveband that commonly features in vegetation indices, including normalized vegetation index (NDVI), enhanced vegetation index (EVI), optimized soil-adjusted vegetation index (OSAVI), green normalized vegetation index (GNDVI), normalized difference red-edge (NDRE), and normalized water difference index (NDWI) align with individual wavelengths in the synthetic traits for Narea (S2–811 nm), PLSR-Narea (S3–808 nm), and PLSR-SLA (S2–808 nm). Likewise, the red waveband that features in NDVI, EVI and OSAVI aligns with one of the wavelengths (743 nm) in synthetic trait S2 for Narea and S3 for PLSR-Narea. For a list with more biologically relevant regions previously published overlaping with the synthetic traits identified see Table S1.

### Future implications

In the past, high-throughput phenotyping data were utilized differently, such as generating vegetation indices from a few wave bands (Da Silva et al. 2020; Crain et al. 2018; Sun et al. 2017; Juliana et al. 2018), partial least square regression models (Yendrek et al. 2017; Weber et al. 2012), NIRs spectroscopy and chemometrics (Ahmed et al. 2024). This usage of high-throughput phenotyping data restricted the exploitation of complete information from the spectra. However, the measurement context and the resolution of spectral data collected at different levels, e.g.., leaf, plot, and field levels, can possess different applications and the nature of information (Barker et al. 2016).

It is important to mention, that while all scenarios of CV2 with synthetic traits were optimistic, some improvements were small and may not represent a practical significance. However, the fact that a synthetically created trait with no intrinsic biological value can improve prediction for a target trait instigates the development of other traits that move beyond linear combinations such as ratios and can potentially result in greater improvements. While we used proximally collected sensor data on the leaf level, there is still a gap in the literature for remotely collected sensor data used in generating the secondary traits for the MT-GP model. The approach of identifying secondary traits using co-heritability measures demands further study on using field-level HTP data integrated into multiple environment multi-trait models.

The method presented here is demonstrated on point data (i.e., no images or timecourses) of hyperspectral reflectance spectra. However, the approach is general in nature and should be easily transferrable to any multidimensional dataset that is gathered alongside target traits. Potential application spaces include remote sensing imagery from UAVs (Varela et al. 2025), controlled environment phenotyping facilities (Schuhl et al. 2026), microscopy images Xie et al. (2021), metabolomics (Aharoni et al. 2023) and ionomics (Baxter 2009). Finally, we observed that similar $coh^2$ values may result in different predictive ability ranges across different MT-GP models. Thus, further investigation on what characterizes the best secondary trait for multi-trait models is warranted.

## Conclusions

This study demonstrated the usefulness of highly heritable correlated traits without any a priori biological value in a GP model to enhance the predictive ability of a target trait. The co-heritability provided us with an efficient option for selecting secondary traits, allowing the identification of helpful information within the spectra. Furthermore, the MT-GP model, which included HTP traits, performed better than the single-trait GP model. These findings are a harbinger for future applications of hyperspectral-based synthetic traits for predicting complex traits.

## Supplementary Information

Below is the link to the electronic supplementary material.

Supplementary file 1 (pdf 2191 KB)

## Data Availability

The hyperspectral data set used in this study is available at https://doi.org/10.5281/zenodo.17280330. All the remaining data are referenced in the methods section.

## Abbreviations

GP: Genomic prediction
MT-GP: Multi-trait genomic prediction
ST: Synthetic traits
HTP: High-throughput phenotyping
NDVI: Normalized difference vegetation index
BLUEs: Best linear unbiased estimates
PLSR: Partial least squared regression
CV: Cross-validation
HPC: High-performance computing
SAVI: Soil adjusted vegetation indices
SLA: Specific leaf area
Narea: Nitrogen leaf area
EF: Energy farm
MW: Maxwell farm
GBLUP: Genomic best linear unbiased predictor
GEBV: Genomic estimated breeding value
Coh$^2$: Co-heritability
ML: Machine learning
NIRS: Near-infrared spectrum

## Code availability

The R package SyntheticTraits devel oped here is available at https://github.com/fernandes-lab/SyntheticTraits. A repository containing all the scripts and documentation on reproducing the results is available at https://github.com/fernandes-lab/Upadhyay2026.
