# Supplementary material for: Improving multi-trait genomic prediction using synthetic traits from hyperspectral data based on co-heritability
Source: Theor Appl Genet. 2026 Sep 18;139(10):270. doi: 10.1007/s00122-026-05334-2 (PMC13588931; doi:10.1007/s00122-026-05334-2)
Supplement: Supplementary file 1 [file 122_2026_5334_MOESM1_ESM.pdf]

Appendix

Table S1: Selected synthetic wavelength ratios and their potential biological relevance.

Target trait	Synthetic trait	Numerator	Denominator	Biological relevance to traditional wavelength
Narea	S1	1677	1716	SWIR (leaf biochemistry such as lignin and cellulose; (Liu et al. 2021) Curran, 1989.
Narea	S2	743	811	Red-edge region (related to strong chlorophyll absorption and internal leaf structure; Horler et al., 1983).
Narea	S3	1077	389	Numerator in SWIR and denominator in visible range, associated with biochemical cell features (Falcioni et al. 2023).
SLA	S1	1719	1680	SWIR (leaf biochemistry such as lignin and cellulose; Curran, 1989; (Liu et al. 2021; Falcioni et al. 2023).
SLA	S2	760	769	Red to near-infrared transition; nearby wavelengths have been associated with chlorophyll content changes (Yoder & Pettigrew-Crosby, 1995).
SLA	S3	1684	1666	SWIR (leaf biochemistry such as lignin and cellulose; Curran, 1989; (Liu et al. 2021) .
PLSR-Narea	S1	1076	389	Numerator and denominator in SWIR and visible regions, highly related to plant metabolic activity (Falcioni et al. 2023)
PLSR-Narea	S2	1337	1730	SWIR (leaf biochemistry such as lignin and cellulose; Curran, 1989(Liu et al. 2021) .
PLSR-Narea	S3	808	743	Near red-edge region; sensitive indicator of healthy green leaf status (Xue et al. 2004).
PLSR-SLA	S1	1728	1661	SWIR (leaf biochemistry such as lignin and cellulose; Curran, 1989; (Liu et al. 2021) .
PLSR-SLA	S2	759	808	Near-infrared region; used for monitoring crop N status (Xue et al. 2004).
PLSR-SLA	S3	388	1088	Numerator in visible range and denominator in SWIR region, related to biochemical cell features.

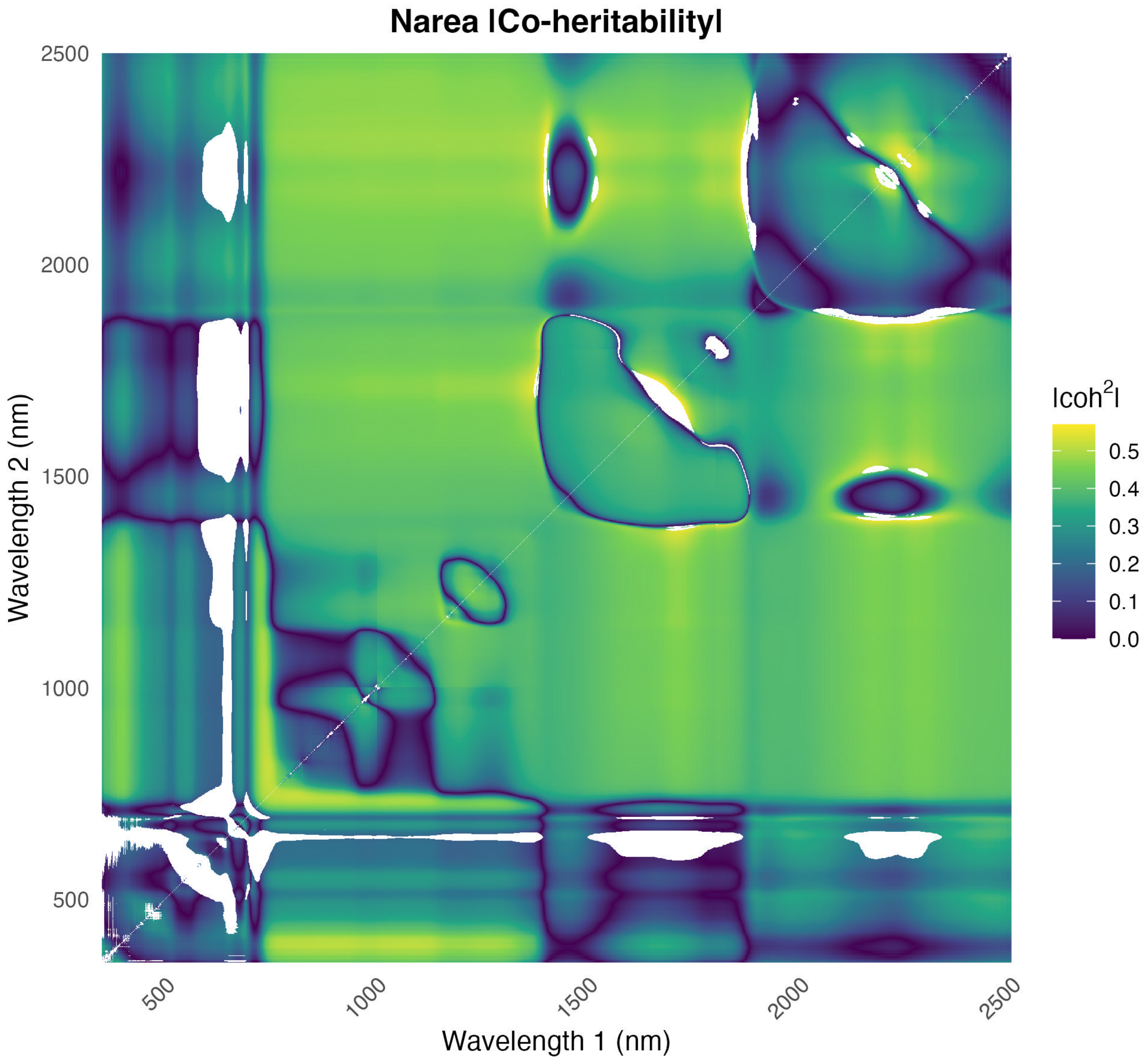

Fig. S1: The heatmap illustrates the absolute value of the coheritability across and between wave-ratios for Narea, with wave1 (y-axis) and wave2 (x-axis) ranging from 350 to 2500 wavenumbers. The color gradient represents coheritability values, where darker shades indicate higher coheritability. The white regions indicate areas where the model failed to converge or was filtered during quality control.

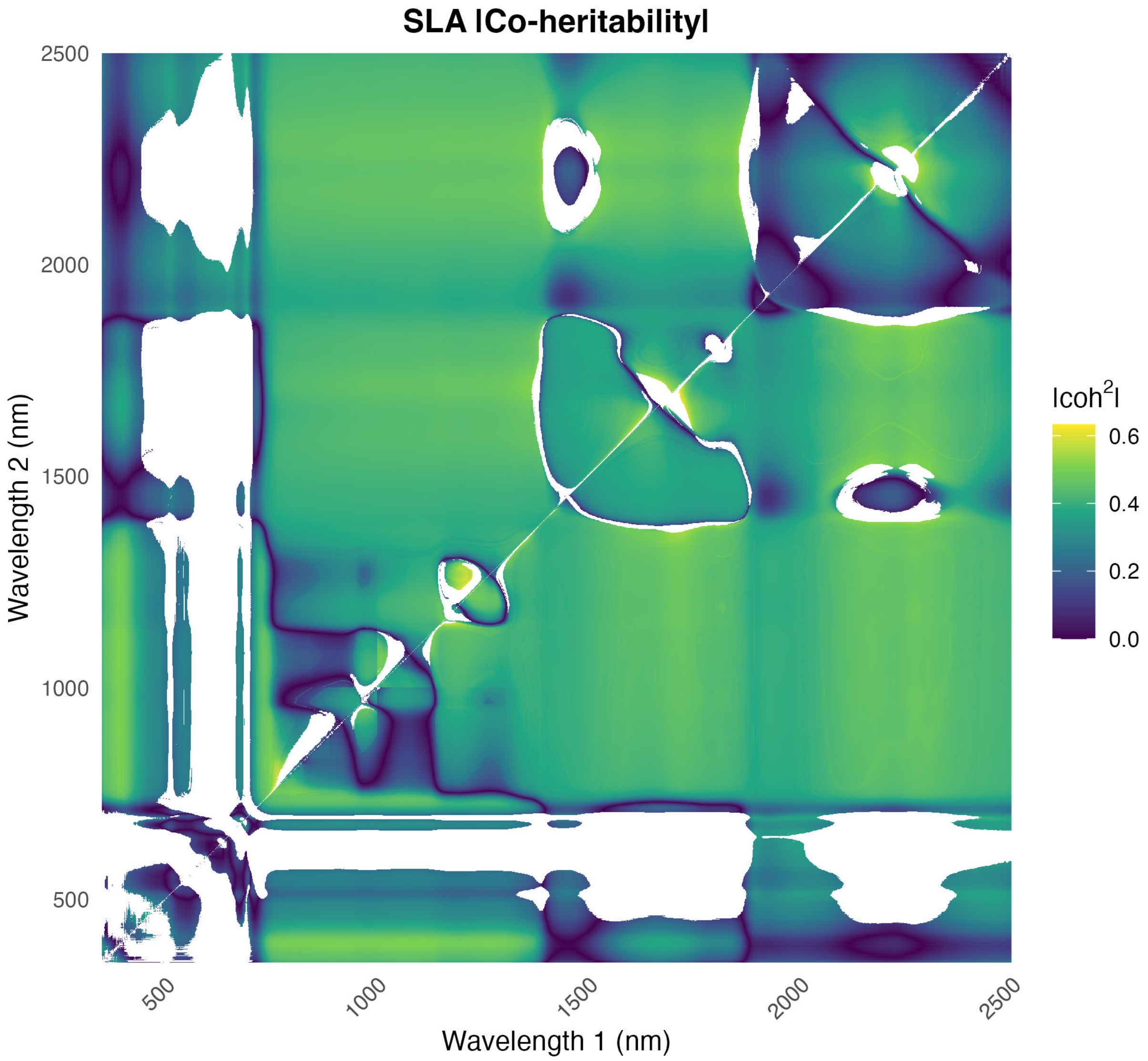

Fig. S2: The heatmap illustrates the absolute value of the coheritability across and between wave-ratios for SLA, with wave1 (y-axis) and wave2 (x-axis) ranging from 350 to 2500 wavenumbers. The color gradient represents coheritability values, where darker shades indicate higher coheritability. The white regions indicate areas where the model failed to converge or was filtered during quality control.

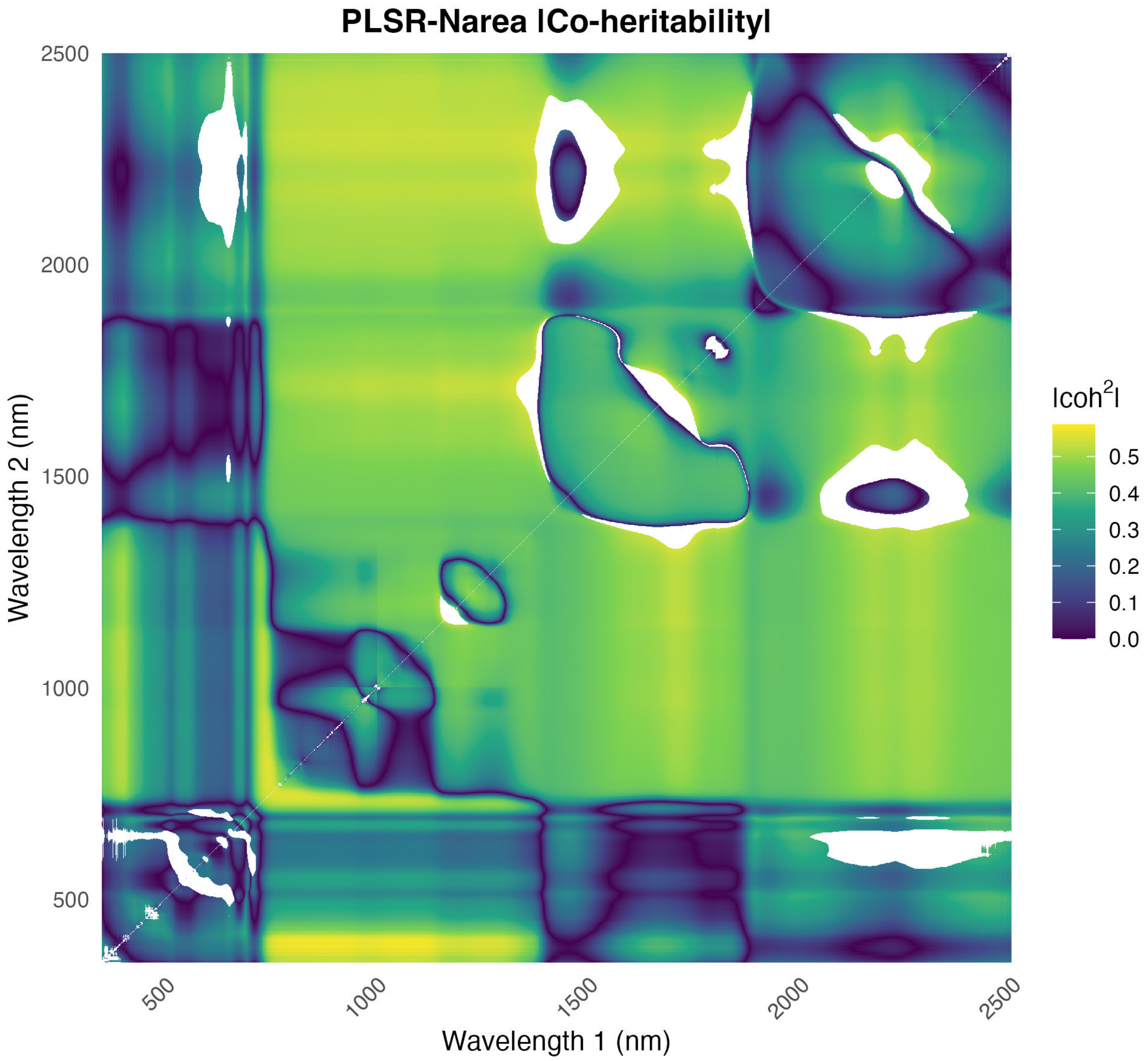

Fig. S3: The heatmap illustrates the absolute value of the coheritability across and between wave-ratios for PLSR-Narea, with wave1 (y-axis) and wave2 (x-axis) ranging from 350 to 2500 wavenumbers. The color gradient represents coheritability values, where darker shades indicate higher coheritability. The white regions indicate areas where the model failed to converge or was filtered during quality control.

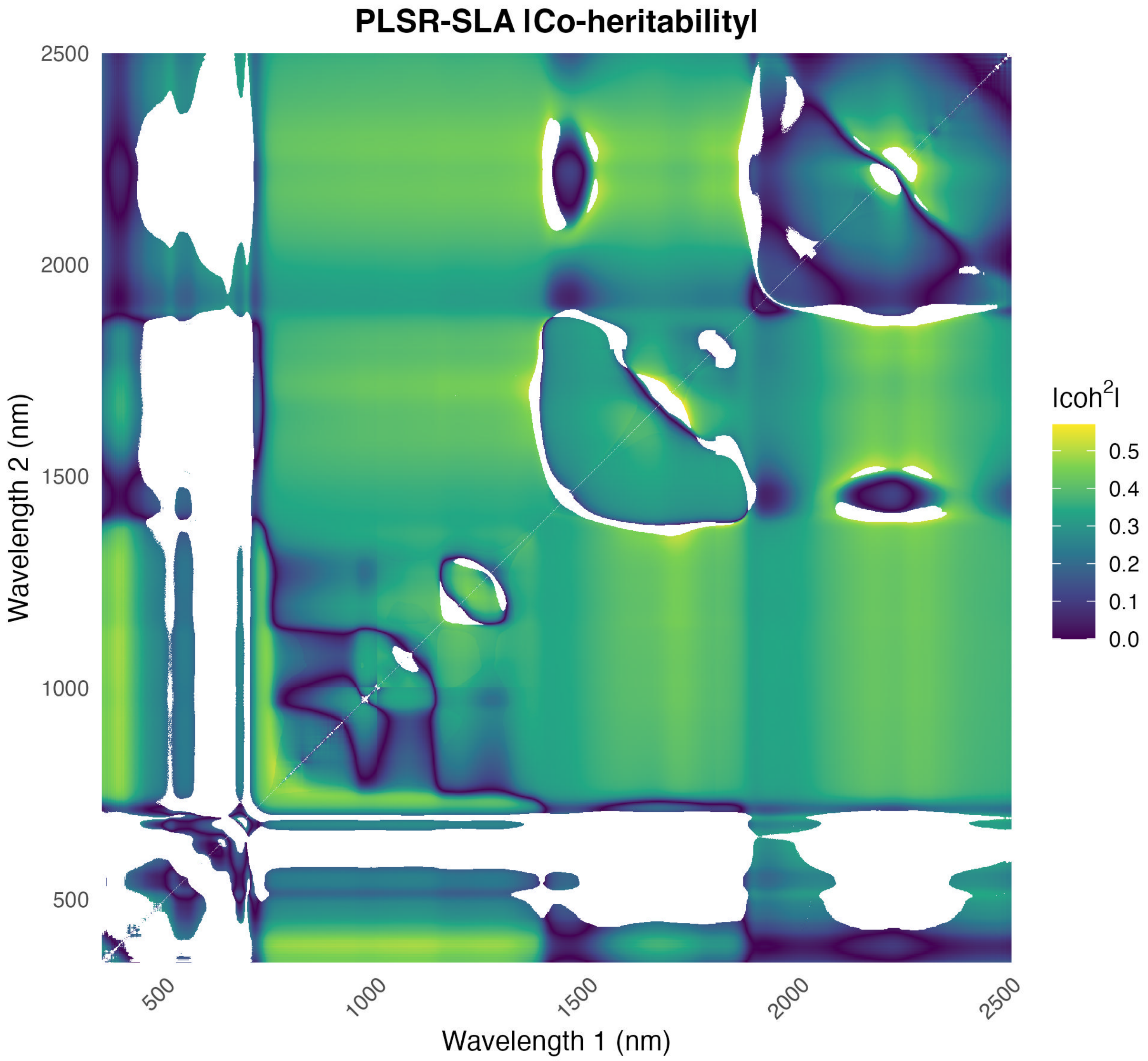

Fig. S4: The heatmap illustrates the absolute value of the coheritability across and between wave-ratios for PLSR-SLA, with wave1 (y-axis) and wave2 (x-axis) ranging from 350 to 2500 wavenumbers. The color gradient represents coheritability values, where darker shades indicate higher coheritability. The white regions indicate areas where the model failed to converge or was filtered during quality control.

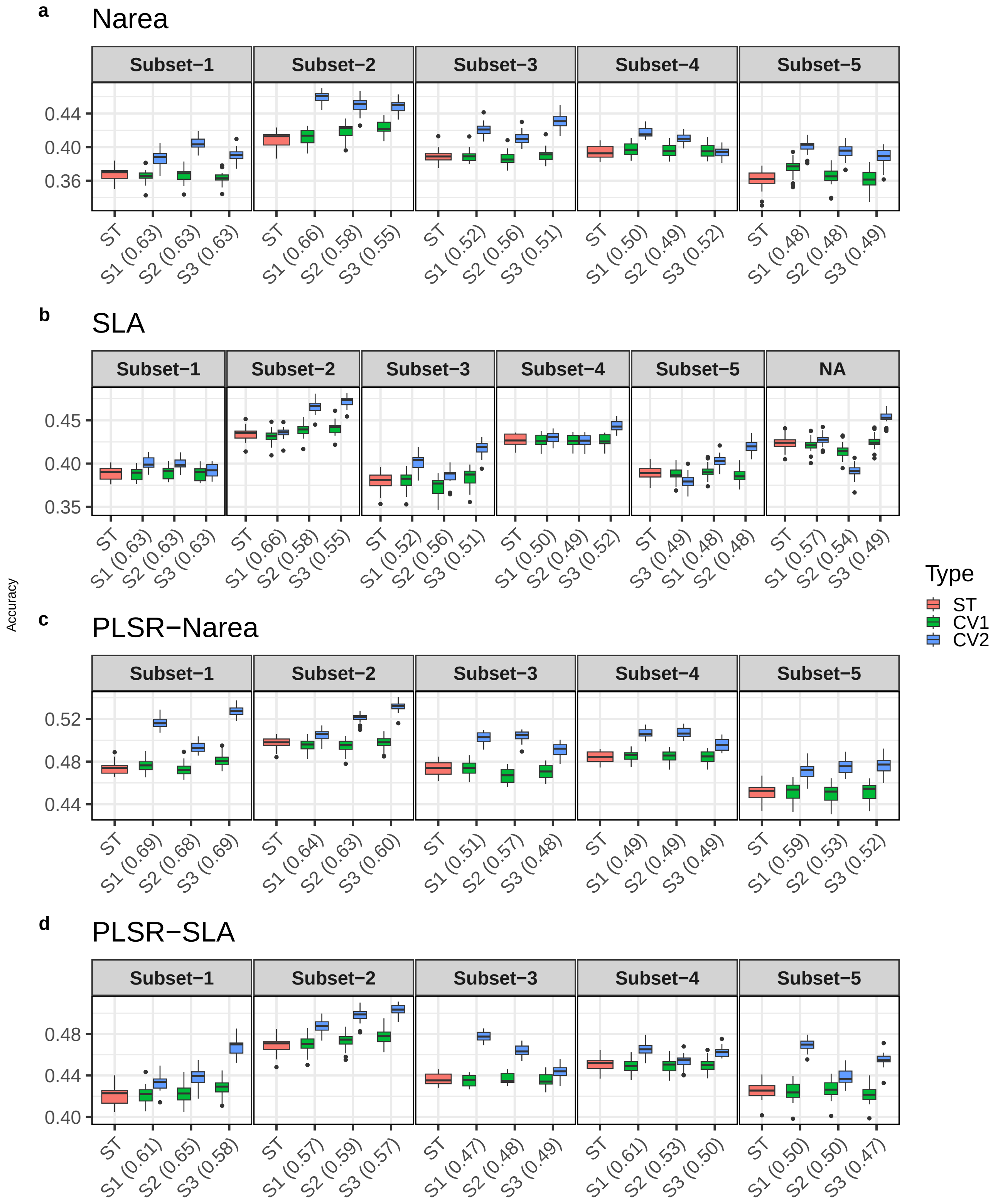

Fig. S5: Comparison of multi-trait (MT) and single-trait (ST) models using synthetic traits identified from subsets of 20% of the individuals. The Y-axis is the predictive ability of models, and the X-axis is the type of model used, i.e., MT and ST. The blue color boxplot is the distribution of predictive ability of 20 replications for CV2, the green color boxplot is for CV1, and the red color is for a single trait. The numbers in parenthesis are the co-heritability values for each synthetic trait. The model was trained using the data set from Maxwell Farm and validated on the data set from Energy Farm.

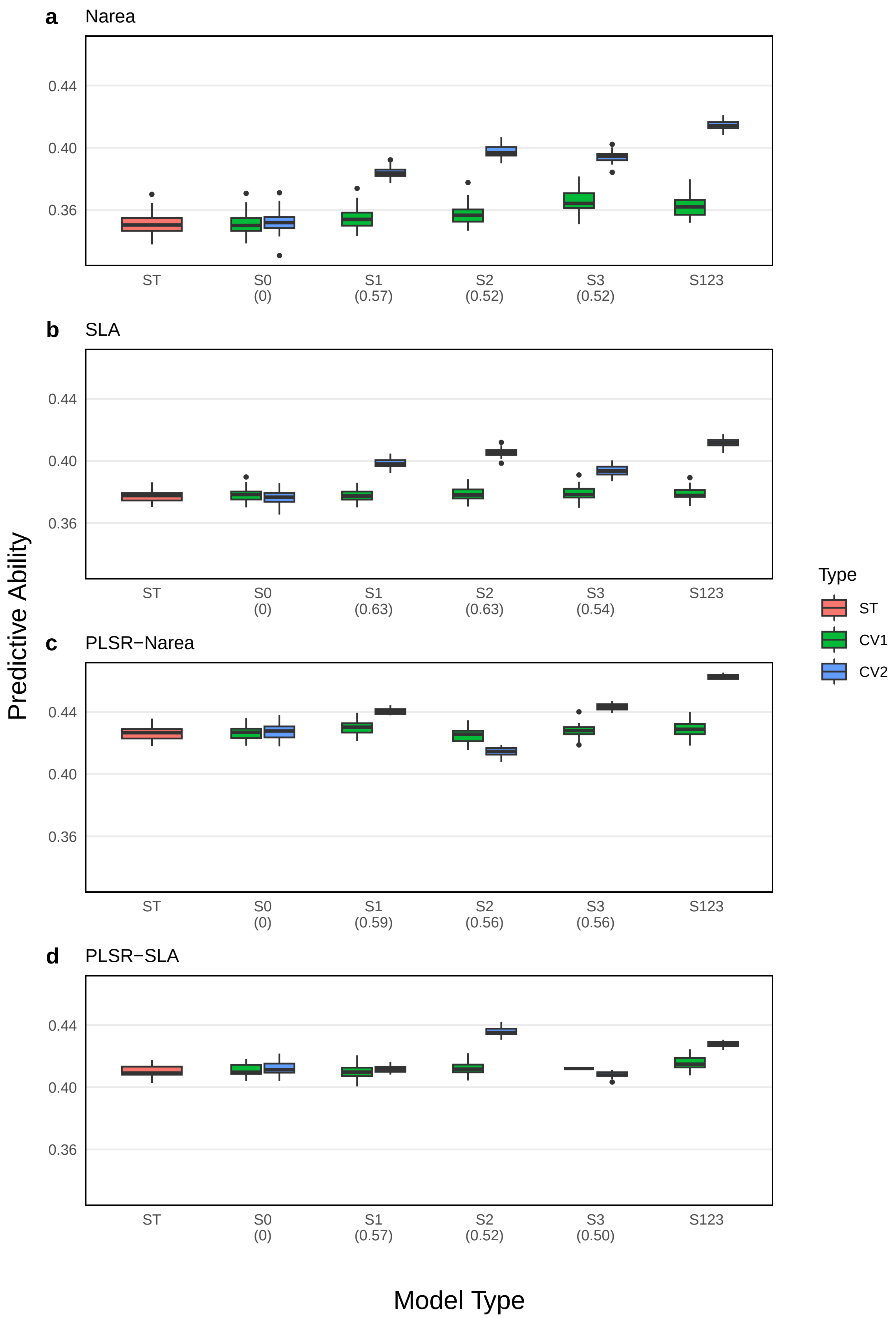

Fig. S6: Single-trait and multi-trait genomic prediction models for four target traits: Narea, SLA, PLSR-Narea, PLSR-SLA. The Y-axis represents the predictive ability for single trait (ST), multi-trait prediction utilizing a synthetic trait with zero co-heritability (S0), Synthetic Trait 1 (S1), Synthetic Trait 2 (S2), Synthetic Trait 3 (S3), and a multi-trait model with the target trait and the three synthetic traits simultaneously. The annotations on the x-axis below the synthetic trait are the coh^2 value for each synthetic trait. The model was trained using the data set from Maxwell Farm (MW) and validated on the data set from Energy Farm (EF). The blue boxplot is the distribution of accuracies for 20 replications for CV2, the green box plot is for CV1, and the red color is for the single-trait model.
